# Weakly-supervised deep learning models enable HER2-low prediction from H &E stained slides

**DOI:** 10.1186/s13058-024-01863-0

**Published:** 2024-08-19

**Authors:** Renan Valieris, Luan Martins, Alexandre Defelicibus, Adriana Passos Bueno, Cynthia Aparecida Bueno de Toledo Osorio, Dirce Carraro, Emmanuel Dias-Neto, Rafael A. Rosales, Jose Marcio Barros de Figueiredo, Israel Tojal da Silva

**Affiliations:** 1 Laboratory of Computational Biology and Bioinformatics, CIPE/A.C.Camargo Cancer Center, São Paulo, São Paulo 01508-010 Brazil; 2 Department of Pathology, CIPE/A.C.Camargo Cancer Center, São Paulo, São Paulo 01508-010 Brazil; 3 Laboratory of Genomics and Molecular Biology, CIPE/A.C.Camargo Cancer Center, São Paulo, São Paulo 01508-010 Brazil; 4 Laboratory Medical Genomics, CIPE/A.C.Camargo Cancer Center, São Paulo, São Paulo 01508-010 Brazil; 5 Division of Cancer Biology, Department of Radiation Oncology, Rutgers New Jersey Medical School, Newark, NJ 07103 USA; 6 Departamento de Computação e Matemática, Universidade de São Paulo, Ribeirão Preto, São Paulo 14040-901 Brazil; 7 Institute of Mathematics and Computer Sciences, Universidade de São Paulo, São Carlos, São Paulo 13566-590 Brazil

**Keywords:** Breast cancer, HER2, Artificial intelligence, Digital pathology

## Abstract

**Background:**

Human epidermal growth factor receptor 2 (HER2)-low breast cancer has emerged as a new subtype of tumor, for which novel antibody–drug conjugates have shown beneficial effects. Assessment of HER2 requires several immunohistochemistry tests with an additional in situ hybridization test if a case is classified as HER2 2+. Therefore, novel cost-effective methods to speed up the HER2 assessment are highly desirable.

**Methods:**

We used a self-supervised attention-based weakly supervised method to predict HER2-low directly from 1437 histopathological images from 1351 breast cancer patients. We built six distinct models to explore the ability of classifiers to distinguish between the HER2-negative, HER2-low, and HER2-high classes in different scenarios. The attention-based model was used to comprehend the decision-making process aimed at relevant tissue regions.

**Results:**

Our results indicate that the effectiveness of classification models hinges on the consistency and dependability of assay-based tests for HER2, as the outcomes from these tests are utilized as the baseline truth for training our models. Through the use of explainable AI, we reveal histologic patterns associated with the HER2 subtypes.

**Conclusion:**

Our findings offer a demonstration of how deep learning technologies can be applied to identify HER2 subgroup statuses, potentially enriching the toolkit available for clinical decision-making in oncology.

## Background

Breast cancer (BC) is the most frequently diagnosed malignancy in women and the second most common cause of death from cancer among women worldwide [1]. BC is a heterogeneous disease with two main morphological subtypes: ductal carcinoma *in situ* (DCIS) and invasive ductal carcinoma (IDC). The latter accounts for 70–80% of cases [2]. The morphological assessment of BC is perhaps the main prognostic factor and is still indispensable in routine practice [3]. The additional evaluation of the estrogen (ER) and progesterone (PR) hormone receptors coupled with human epidermal growth factor receptor 2 (HER2) categorizes BC into the following clinically relevant immunophenotypes: (i) luminal A (LUMA), (ii) luminal B (LUMB), (iii) triple-negative breast cancers (TNBC), and (iv) HER2-enriched (HER2+).

The assessment of the proliferative index (Ki-67) together with ER, PR, and HER2 status is currently determined by immunohistochemistry (IHC)-based methods followed by visual evaluation by a pathologist [3]. Several research efforts have explored gene expression-based signatures for specific BC stratification [4], informing the selection of precisely targeted intervention therapies. For example, a clinically applicable gene expression assay using 50 genes (PAM50) that classifies breast cancer into four molecular intrinsic subtypes shown above has been reported [5].

In particular, the HER2 assessment has been reshaped by considering the first Food and Drug Administration approved targeted therapy for a new classification of the HER2 subtype called HER2-low [6]. Usually, the assessment of HER2 protein overexpression on the surface of cells is evaluated by IHC using a well known four-tier scoring system of 0 to 3+ (0: negative; 1+: negative; 2+: equivocal and 3+: positive) [7]. The HER2-low subtype includes tumors with IHC scores of 1+ or 2+ without amplification by an additional in situ hybridization (ISH)-based test [6]. Although the 2+ assessment by ISH is the most accurate, it is more expensive, and obtaining the results takes more time, thus remaining a bottleneck step in current clinical practice in most hospitals [8]. This justifies efforts for the development of novel approaches that aim to quantify HER2-low and should benefit patients with a faster and more accurate classification method that could better guide therapeutics.

Although the assessment of most biomarkers in BC is based on expanded IHC and gene expression profiling analyses, recent advances in deep learning (DL) have the potential to complement traditional assays [9]. Motivated by the ability to learn from underlying features directly from hematoxylin and eosin (H &E) stained histopathological whole slide images (WSIs), we and others have developed DL-based systems to predict diverse molecular phenotypes including mutations in non-small cell lung [10], microsatellite instability (MSI) in patients with gastrointestinal cancer [11], homologous recombination deficiency (HRD) [12] and other clinical biomarkers [13]. These studies further emphasize the ability of machine learning methods to learn underlying morphologic features that mirror the expression of molecular markers.

Although the relevance of machine learning for BC applications has been investigated [14–21], most models have been trained on images of tissue microarrays, IHC-stained slides and fluorescence in situ hybridization (FISH)-images. Furthermore, the classifiers were used to predict HER2 either within the hormone receptor status or intrinsic subtype, in which HER2 status was classified solely as positive or negative. Most importantly, in light of treatment options available for HER2-low patients, the assessment of HER2-low tumors by DL models remains to be explored.

In this study, we built a series of deep learning models to examine different training datasets by combining quantitative scores from either IHC tests or ISH assays for HER2-low assessment in BC patients. Moreover, we explored the potential of explainable AI (xAI) mechanisms for providing insights into the underlying causes of the model’s decisions. Lastly, we assembled a novel online dataset, containing more than 2 million H &E image patches.

## Methods

In total, data were gathered from 1437 H &E-stained WSI of 1351 patients across three multicentric cohorts. These included an in-house cohort (ACCCC, N=546) and two public repositories (TCGA, N=535 and HEROHE, N=356).

### ACCCC cohort

We used an anonymized dataset from patients treated for BC at A.C. Camargo Cancer Center (ACCCC, N = 504) between 2019 and 2021. The HER2 tumor status within the ACCCC cohort was determined in accordance with standard procedures outlined by the American Society of Clinical Oncology/College of American Pathologists (ASCO/CAP) guidelines. In total, we obtained and digitized 546 H &E-stained WSI at 0.25 ${\upmu }$m/px magnification from both biopsy and resection tissue samples using a Leica Aperio AT2 scanner. This study received ethical approval (#3028/20) from the ethics committee of the Fundação Antônio Prudente, with all cases with metastasis or neoadjuvant treatment were excluded. Supplementary Table 2 presents the detailed histological characteristics of our in-house cohort.

### HERO cohort

The HERO dataset was downloaded from a publicly available database, published by The HEROHE Challenge [22, 23], which aimed to create new tools for predicting HER2 status in H &E-stained WSI from biopsy samples of patients with invasive breast cancer. The WSI are available at https://ecdp2020.grand-challenge.org. These slides were scanned at Ipatimup Diagnostics using a 3D Histech Pannoramic 1000 digital scanner at 0.25 ${\upmu }$m/px magnification, and saved in the MIRAX file format [23]. The HERO dataset is composed of 509 slides from distinct patients and after an initial background tile filtering were reduced to 356 slides. Details about the distribution of IHC scores and HER2 status are summarized in Table 1, which were classified by experienced pathologists according to the latest ASCO/CAP guidelines [23].

### TCGA-BRCA cohort

A total of 1072 H &E stained WSI at 0.25 ${\upmu }$m/px magnification from 1063 patients was retrieved from the GDC portal TCGA-BRCA project, which contains clinical information and is available at https://portal.gdc.cancer.gov/. Only high-resolution H &E stained WSI were selected for analysis, excluding those with artifacts such as significant pen marks, tissue folds, or blurriness, we also only selected slides that had HER2 test results available in the clinical data, after filtering a total of 535 slides remained for the analysis. The HER2 status in TCGA was determined using both the IHC score and FISH status.

**Table 1:** Distribution of slides across the three cohorts and HER2 groups

Cohort	HER2-neg	HER2-low	HER2-high	Total
ACCCC	196	217	133	546
HERO	23	187	146	356
TCGA-BRCA	40	377	118	535
Total	259	781	397	1437

### Ground truth for HER2 status

All models were trained with ground truth data derived from a combination of IHC tests and DDISH/FISH tests. Specifically, the HER2 protein expression is initially assessed using an immunohistochemistry assay, which involves staining breast cancer tissue samples with antibodies specific to HER2. The staining intensity and pattern are then evaluated by a pathologist resulting in four possible scores (0: negative; 1+: negative; 2+: equivocal and 3+: positive). In case of an equivocal result, an additional in-situ hybridization test is performed either by Fluorescence in situ hybridization (FISH) or dual-color dual-hapten brightfield in situ hybridization (DDISH).

For the purposes of our study, all cases were subdivided into three groups according to their IHC score and ISH status: HER2-neg, HER2-low, and HER2-high, as described in Table 2.

### Data preprocessing

In the pre-processing phase, we developed a model to remove background and detect tumor regions, aiming to segment informational tissue regions from the WSI. We employed the following procedures to achieve this goal. First, we trained a ConvNeXt-Tiny model [24] with the *NCT-CRC-HE-100K* dataset [25], which contains examples of background tiles. Applying this model on the TCGA-BRCA, we constructed a set of background region annotations. Next, we used annotations from the Manual Tumor annotations dataset [26] to create a set of non-tumor annotations from the regions in the slide that are neither tumor nor background. Finally, we trained a combined model (tumor-background) on the entirety of TCGA-BRCA slides to identify tumor, non-tumor, and background regions based on these annotation sets. The prediction of the tumor-background model is illustrated in Supplementary Figure 1.

To process the slides prior to the MIL model training, each slide was divided into 256 px $\times$ 256 px tiles at 0.5 ${\upmu }$m/pixel magnification, if the original slide had higher magnification a corresponding down-scaling transformation was applied, with adjacent tiles having a 50% overlap. Next, the tumor-background model was used to detect background regions, tumors, and non-tumors in all cohorts. Only tiles identified as tumor were used to train the MIL model, slides with less than 1000 tumor tiles detected were excluded from further analysis.

### Feature extraction

Following the color normalization procedure by [27], we normalized slides from all cohorts to a reference image from the ACCCC dataset. Next, a 1024-feature embedding vector was extracted from each color-normalized tile by using the *UNI* model. This is a general-purpose self-supervised model for pathology, trained with more than 100 million images from over 100,000 diagnostic H &E-stained WSIs across 20 major tissue types [28]. The 1024-feature vector was used in the following steps to train the MIL model.

### MIL model definition

We use a weakly supervised multiple instance learner (MIL) classifier, which takes an H &E slide in the format of a bag-of-tiles as input data and classifies it into one of the desired classes using an attention-based mechanism. The MIL architecture considered throughout was based on clustering-constrained-attention multiple-instance learning (CLAM) [29].

Briefly, the CLAM model considers the features of a slide as a bag of tiles and classifies the slide into one of the target classes. Internally, it works by modeling each HER2 group independently with an attention layer and a classification layer. The attention layer is responsible for identifying important tiles and combining them into a single 1024-feature vector for the slide based on the perceived importance of its tiles, while the classification layer takes this representation vector and outputs a single activation value: higher outputs represent a higher likelihood of the slide belonging to the class. Finally, this independent value for each HER2 group is then combined in the output vector using a *softmax* activation function, whose maximum value describes the slide’s HER2 group. Additionally, the model also employs an *instance classifier* whose goal is to cluster the tiles of each class. During training, the *instance classifier* acts as a regularization method that biases the attention backbone into producing relevant features for both the attention mechanism and itself; however, once the model completes the training stage, it can be used as a tile classifier that identifies tiles that are associated with its target HER2 group.

### Model training

The ACCCC and HERO cohorts were combined into a single dataset and 15% of the cases were separated for a hold-out test set. The remaining 85% of the cases were further split into 10 cross-validation folds where cases were randomly assigned to a training (85%) or validation set (15%) while ensuring that the label distribution and slide dataset proportion of both sets remained unchanged. All models were trained independently for each fold using the same set of fixed hyper-parameters with the goal of evaluating the model variance on different data. The TCGA-BRCA cohort was used as an external test dataset.

We tested six models, each considering a different subset of cases with assay results of HER2 status analyzed at the protein level by IHC and ISH techniques (Table 2). Each model was trained multiple times with a 10-fold cross-validation procedure. All models were trained for a total of 200 epochs with the early stopping criterion set to the loss of the validation set. To speed up training and diminish GPU memory requirements, we divided the training procedure into two stages: i) feature extraction and ii) CLAM classifier training. This approach has the advantage of allowing more models to be trained in parallel due to the lower memory and processing requirements of training each model. All models were trained using an NVIDIA A100 40 GB GPU.

We employed the CLAM’s originally proposed loss function as the optimization objective, which is a two-part linear combination consisting of a ’bag’ loss and an ’instance’ loss. The ’bag’ loss, a Cross-Entropy loss, is weighted by 0.7, while the ’instance’ loss is a Smooth SVM Loss. The training process utilized the ADAM optimizer with a fixed learning rate of 0.0001 and weight decay of 0.0001. The resulting model has 2.1M parameters.

### Analysis of actionable tiles

Actionable tiles can be identified by the CLAM’s instance layer, which is a set of binary classifiers available for each class (in our case, HER2 groups). Its goal is to identify whether a given tile belongs to its specific class. For each slide in the internal test set, we classify the tiles with the instance layer, selecting those with a predicted probability above 0.5 for their respective class. The top 15 tiles with the highest attention value of each slide identified by the instance classifier are selected as actionable tiles. In total, we sampled 100,280 actionable tiles across all HER2 groups and models, regardless of the final whole-slide classification. Hence, our goal is to understand what features were learned as being relevant for each group.

To analyze the actionable tiles, we propose a visualization approach based on a 2D histogram of the latent feature space. First, we reduce the dimensionality of the tile feature vector with PCA, projecting the first two principal components of the features onto two dimensions. Then, we discretize this space into 30 $\times$ 30 bins, with each of the bins acting as a centroid around the sampled tile’s embeddings, clustering the tiles with similar features. This approach allows us to characterize the continuous space of tile features by coloring the bins based on different criteria. For instance, bins can be colored using the HER2 group that appears more frequently, or we can color them only when two or more models use their corresponding feature. This approach allows us to organize and understand the space of actionable histological features.

## Results

**Fig. 1:**
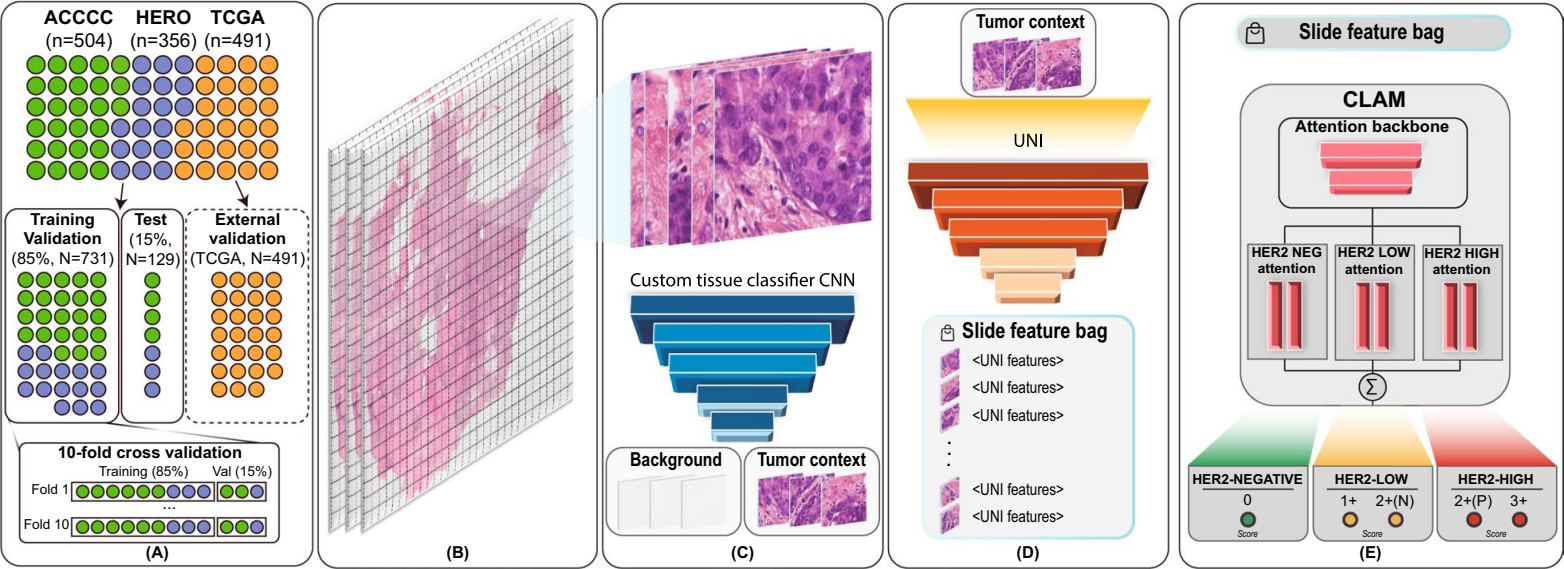
Workflow of the proposed method. **A** Datasets (ACCC, HERO, TCGA) are split by patients (N) into four categories: training and validation (85%, ACCC+HERO); test (15%, ACCCC+HERO); external validation (TCGA). **B** Whole-slide Images are split into tiles; **C** regions of interest are identified; **D** features are extracted from tile images; **E** the CLAM model is trained to classify tile sets into HER2-negative, HER2-low, and HER2-high groups

The purpose of this study was to develop a deep learning model for the assessment of HER2-low tumors. To this end, we develop a computational pipeline involving the following three steps (Fig. 1):

1. Tiles were extracted from whole-slide images (WSI) and subsequently classified into tumors and nontumor groups (Fig. 1B, C).
2. The generated bag of tiles for the tumor and nontumor groups were then used for feature extraction with the UNI model [28] (Fig. 1D).
3. The resulting bags of features were used to train a multiple instance learning (MIL) model for classifying the WSI into HER2-negative, HER2-low, and HER2-high classes (Fig. 1E).

### Model performance reveals that classifying HER2-low is challenging

**Fig. 2:**
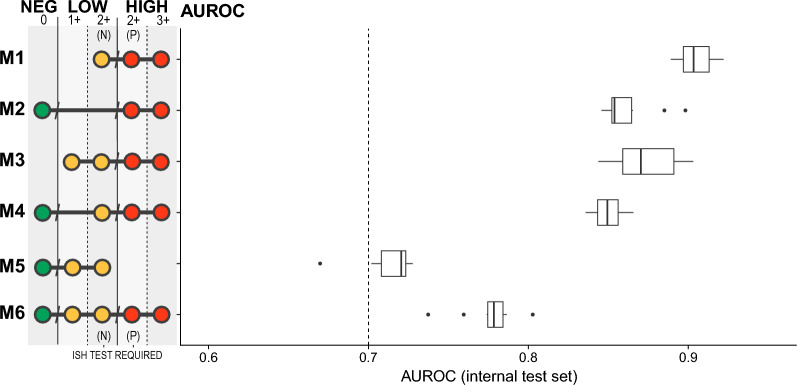
AUROC of the 10-fold cross-validation results for the test set. Each model was trained with its own set of HER2 scores, i.e., each target HER2 group. The HER2 scores used are indicated by the dots, while the dot’s color indicates its target HER2 group for the model

**Table 2:** Data partitioning scheme of each model

	HER2-neg	HER2-low		HER2-high	
IHC	0	1+	2+		3+
ISH			Neg	Pos	
M1			$\bullet$	$\bullet$	$\bullet$
M2	$\bullet$			$\bullet$	$\bullet$
M3		$\bullet$	$\bullet$	$\bullet$	$\bullet$
M4	$\bullet$		$\bullet$	$\bullet$	$\bullet$
M5	$\bullet$	$\bullet$	$\bullet$		
M6	$\bullet$	$\bullet$	$\bullet$	$\bullet$	$\bullet$

Data partitioning scheme of each model. We divided the cases into the following 3 groups according to the results of the IHC and ISH status: HER2-neg, HER2-low, and HER2-high

We built six distinct models to explore the ability of classifiers to distinguish between the HER2-negative, HER2-low, and HER2-high classes in different scenarios. The detailed trained set used in these models is summarized in Table 2 and the performance is shown in Fig. 2. Specifically, while the M6 model was trained to classify the 3 classes with the whole dataset, we also trained models with subsets; for example, M1 was trained with only HER2 2+ and 3+ cases, to detect HER2-low on the HER2 2+ subgroup without the need for a confirmatory additional ISH-based test.

To assess the learning variability of these models we performed a 10-fold cross-validation on each of the subsets tested, the hold-out internal test AUC for each fold can be seen on boxplot variance as shown in Fig. 2. ROC curves for each model and fold are included in the Supplementary Figure 2. Precision-Recall curves are shown in Supplementary Figure 3.

We assessed the detection of HER2-low in HER2 2+ results based on the IHC-based assay results (M1). In this model, the training dataset includes results where there is an inherently subjective and high interobserver agreement, which guarantees a good discriminatory capacity of this classifier. Notably, this model has great potential for use in a cost-effective manner, since HER2 2+ IHC results require confirmation by an additional assay based on hybridization methods. Next, we sought to evaluate the ability to discriminate groups of patients with HER2 0 (negative, scoring 0) vs. HER2 2+ (with scoring 2+ with additional positive ISH results and scoring 3+ being equivalent). In fact, when considering that molecular changes precede and results altered cellular histology, the M2 model was able to detect groups of patients with a basal HER2 expression (without HER2 amplification) as distinct from those with high expression. Therefore, it is reasonable to expect great performance from this model as compared with the former model. Notably, by considering either groups labeled as HER2-low (HER2 1+ and HER2 2+ with additional ISH negative), we noted a performance gain to discriminate HER2-low. However, the M4 and M5 models show a performance drop when we either include the HER2 0 and subtract the HER2-high (HER2 2+ with additional ISH positive and HER2 3+) groups. Although we are using labels ground truth results from well-standardized clinical practice, these results suggest a possible confounding effect on the trained classes when considering the HER2 1+ group. This is a result of the subjectivity and slight difference in the results (low interobserver agreement) obtained from the IHC assays by the experts of the HER2 0 and 1+ groups [30], which consequently affects the performance of the models since the results of the molecular tests are considered in the division of the training and testing groups of the models. Although building a model to classify samples into 3 groups is challenging, we observed that the M6 (ideal) model shows a discriminatory power to predict HER2 group directly from H &E histology. The worst performance is obtained from the fM5 model, which must detect HER2-low type tumors among the HER2 0, 1+, and 2+ groups with negative ISH results; ultimately, this demonstrates a limitation of the model when considering results from assays where there is discordance in distinguishing HER2 0 from HER2 1+ [30, 31].

Importantly, HER2 assessment protocols are continuously updated according to the recommendations of the American Society of Clinical Oncology/College of American Pathologists (CAP) [32] and thus gathering high-quality datasets with consistent labeling of the HER2 groups (neg, low and high) is challenging, this hampers the evaluation of the models capability to generalize to independent datasets. Indeed, we used the TCGA breast cancer data for HER2 prediction as an external cohort. In particular, we observed the same trend for all models built considering the distinct datasets (Supplementary Table 1, Supplementary Figure 4). However, since the TCGA-BRCA dataset used a different set of guidelines for HER2 assessment, we observed a drop in performance for all models, as expected. Furthermore, the TCGA database aggregates image datasets from numerous institutions, each employing varied protocols for sample preparation and image capture. Such technical diversity also affects classification performance [19].

Altogether, our findings suggest that the performance of classifiers is dependent on the reproducibility and reliability of assay-based tests for HER2 since the results of these assays serve as the ground truth for model training.

### Actionable tiles reveal patterns used by the classifier

**Fig. 3:**
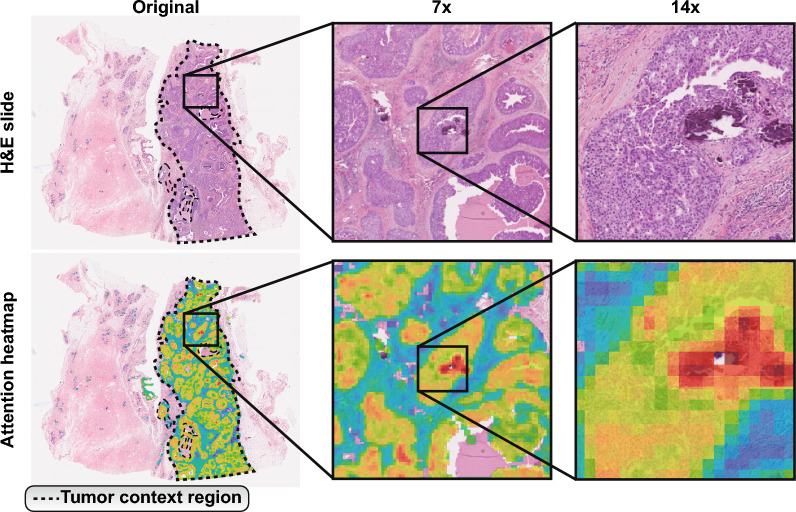
H &E-stained slide of a tissue sample and its corresponding attention heatmap generated by the proposed model. Attention maps highlight meaningful patches at different magnifications. The heatmap shows the areas where the model focuses more of its attention, with warmer colors indicating higher attention scores. The dotted line shows the region identified as tumor

A key step in deep learning in a clinical setting is interpreting how the generated models work, thus allowing us to evaluate their validity and trustworthiness. To understand which histological patterns are used by the model to classify the HER2 classes, we need to examine which features are being used by the MIL classifier. To this end, we considered the attention layers provided by the MIL algorithm [33]. These layers direct the classifier to a subset of tiles with features that allow for the classification (Fig. 3). We focus our analyses on tiles that are given high levels of attention, denoted as actionable tiles, due to their importance on the final result of the classifier.

**Fig. 4:**
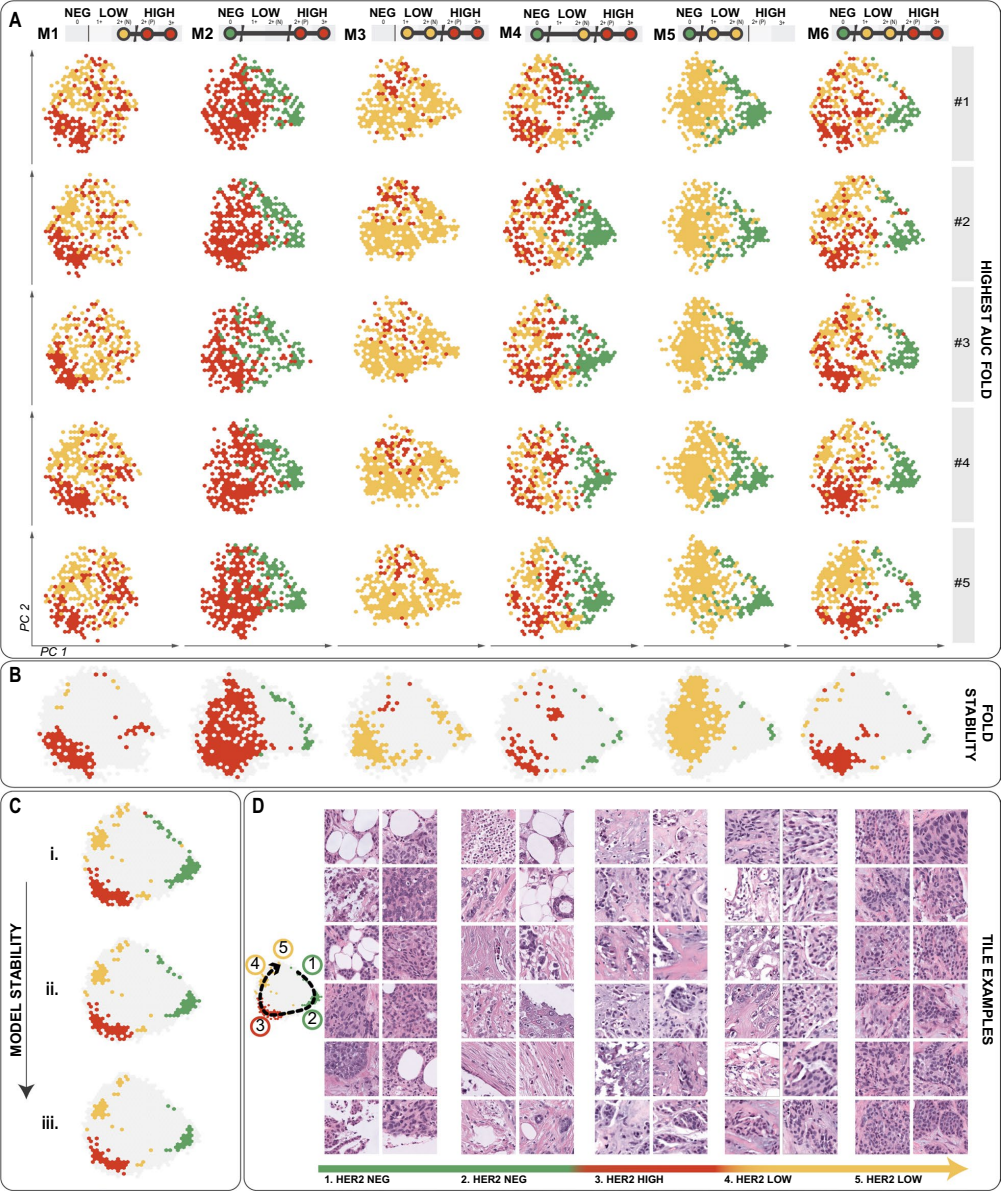
Model explicability analysis. **A** Tile embedding is plotted using the PCA results for each model and fold, only coloring regions according to the HER2 group classification that relies on attention to that embedding. **B** Colored bins indicate features used by 50% of the folds within each model. **C** The consistency of the models highlights how many models use the same embedding for classifying each HER2 group. There are three levels of agreement: i. 2 or more; ii. 3 or more; iii. 4 or more. **D** Examples of tiles from the embedding space for each class

The resulting representation revealed patterns across different models and inside the folds for the same model (Fig. 4). The analysis of different folds by the same model reveals the occurrence of recurring patterns, indicating that tiles of similar features in these areas are being used for the classification of the same class.

Tiles classified into a given HER2 class tend to form clusters on the two dimensional principal component space. Tiles from these clusters share morphological characteristics that define each class. A sample of the image tiles of each such cluster in Fig. 4C–D supports this (with more tiles examples in Supplementary Figure 5).

While histopathological evaluation of tissue sections stained with hematoxylin and eosin (H &E) is indispensable for the management of breast cancer, the relationship between cell pattern and HER2 subgroups is still unclear. We thus examined the cellular patterns of the actionable tiles associated with the HER2 groups. We observed actionable tiles in HER2-high with the presence of hyalinized stroma with minimal inflammatory infiltrate, possible isolated neoplastic cells, and various distinct histologic patterns including cohesively patterned neoplasia with lobular infiltration patterns of nuclear grades 1/2, and structural patterns that resemble micropapillary features. This subtype also exhibited tiles with nuclear grades 2/3 but predominantly grade 2, and rare instances of mitotic activity. Conversely, we noted actionable tiles in HER2-neg presented nuclear grade 3 and were enriched for adipose cells. Importantly, this adipose microenvironment could be associated with motility and invasiveness of breast cancer cells [34]. Reinforcing this observation, we noticed some tiles with muscular tissue infiltrated by isolated neoplastic cells, a feature that can be associated with aggressiveness since muscular infiltration of thoracic wall muscles characterizes advanced-stage tumors. Putting it all together, the presence of apocrine features, higher nuclear grade and a more infiltrative pattern indicate a more aggressive tumor, often associated with triple-negative breast cancers (TNBC). Finally, actionable tiles in HER2-low were enriched for chronic inflammatory cells, fibrotic tissue, and predominantly nuclear grades 1 and 2. These histology findings provide a nuanced understanding of the heterogeneity within breast cancer, underlining the importance of explainable visualizations for elucidating how certain spatial pathology features correlate with HER2 groups and specific diagnoses.

### Filtering tiles of interest guides training

**Table 3:** Median and standard deviation of the AUROC of the internal test

	NOBG	TUMO
M1	0.88 +0.02	**0.90 +0.01**
M2	**0.87 +0.02**	0.85 +0.02
M3	**0.91 +0.02**	0.87 +0.02
M4	0.85 +0.01	0.85 +0.01
M5	0.69 +0.04	**0.72 +0.02**
M6	0.78 +0.01	0.78 +0.02

Median and standard deviation of the AUROC of the internal test set on the 10-fold cross-validation with different tile filtering strategies. The best performance for each model is highlighted in bold

Given a tumor identified as HER2-negative through an IHC stain, it is reasonable to infer that its corresponding H &E WSI lacks discernible features indicative of HER2-positivity. Conversely, in cases where the patient is identified as HER2-positive, it is reasonable to assume that at least certain regions within their H &E WSI exhibit features relevant to HER2-positivity. Consequently, models can be trained using WSI stains as input data and IHC annotations as data labels. Hence, this problem formulation aligns effectively with the principles of multiple instance learning (MIL) [35]. However, as digital slides may incorporate distinctive artifact patterns resulting from the staining protocol or digitalization process, which might be captured by the MIL algorithm, we conduct background detection and removal of tiles from the dataset prior to MIL model training. This is undertaken to mitigate the potential impact of such tiles and minimize any spurious effects they may introduce.

Importantly, one of the most common methods for background removal is a thresholding-based algorithm such as the Otsu method [36]. However, such methods are not able to detect other types of artifacts such as out-of-focus regions and colored pen markings, which are present in some datasets, including TCGA dataset.

To investigate whether the inclusion of specific subsets of tiles impact the results of the MIL model, we developed a neural-network method to identify and filter background, tumor, and non-tumor regions, as described in the Data preprocessing section. We illustrate the effect of this filter by training models with different subsets and evaluating the performance of each model (Table 3, Supplementary Figure 6). First, we attempted to filter only background tiles (NOBG), and we found that the model is able to distinguish tissue-specific patterns; however, we also found that the model learns to give high levels of attention to tiles showing the tumor environment, which prompted us to test the models using only tiles from the tumor region. We refer to this filtering configuration as *tumor only* (TUMO), and we found that by using the tumor environment, we were able to achieve the best balance between model performance and training speed, as the models only need a fraction of tiles (Supplementary Figures 7 and 8) to achieve similar performance to models using the full set (Supplementary Figure 6).

Our analysis indicates that models built only with tumor tiles (TUMO) performed similarly to models that used all tiles except background (NOBG). This suggests that by filtering the tiles, the model can focus on the most important areas, lowering the risk of attending spurious unique patterns such as artifacts or background noise. We chose to use TUMO because it strikes the optimal balance between model predictive performance and hardware requirements.

### Comparison of alternative implementations

**Table 4:** Median and standard deviation of AUROC performance

	ADMIL	CLAM	TransMIL
M1	0.87 +0.02	**0.90 +0.01**	0.88 +0.03
M2	0.85 +0.02	0.85 +0.02	0.85 +0.02
M3	0.84 +0.02	0.87 +0.02	**0.88 +0.04**
M4	*N/A*	0.85 +0.01	*N/A*
M5	**0.73 +0.05**	0.72 +0.02	0.71 +0.02
M6	*N/A*	0.78 +0.02	*N/A*

Median and standard deviation of AUROC performance on the internal test set for ADMIL, CLAM, and TransMIL. ADMIL and TransMIL can only work on binary models. The best performance for each model is highlighted in bold

We compared the results from the CLAM model with two other implementations of the MIL method: ADMIL [33] and TransMIL [37], the results are summarized in Table 4, Supplementary Image 9. The methods presented comparable performance in most models, however CLAM showed better overall performance. Importantly, only the CLAM method permits multi-class predictions, while the ADMIL and TransMIL method implements only binary prediction, thus we choose to employ the CLAM method.

We also compared the use of the *UNI* feature extractor with a *ResNet-50* trained with the ImageNet dataset [38]. Notably, all models trained with *UNI* outperformed models trained with *ImageNet* features (Supplementary Figure 10). This highlights the importance of foundation models trained for a specific domain.

## Discussion

HER2-positive BC is characterized by higher levels of epidermal growth factor receptor 2 (HER2), where anti-HER2 targeted therapies have shown to be effective. More recently, the approval of targeted therapies in HER2-low tumors has expanded the efficacy of BC treatments to benefit countless patients. Herein, we explore the ability to detect HER2 status directly from H &E images using deep learning. We built six models aiming to explore the ability of classifiers to distinguish between HER2-negative, HER2-low, and HER2-high classes from distinct datasets of IHC and ISH-based assays for HER2 status assessment. We show that models trained to distinguish HER2-negative from HER2-low had lower performance compared to those that only classified between HER2-negative and HER2-high or HER2-low and HER2-high. Thus, we argue that the known challenges [7] in distinguishing HER2 score of 0 from 1+ may affect the quality of data needed for training the algorithms. Improvements in the pathologic workflow, including stringent quality controls [30, 39] and novel quantitative testing that allows measuring a range of antibody concentrations may help to maximize the sensitivity of HER2 expression.

On the external test set, we observed an overall drop in performance compared to the validation set, which is expected, considering that the TCGA cohort is a multi-centric heterogeneous dataset. This effect can also be explained as a result of overfitting. Notably, the M2 model performed significantly better on the external test set compared to the other models, suggesting that the distinction between HER2-neg and HER2-high is better defined, and the classification accuracy might more easily be translated to new cohorts.

When selecting which slide tiles to utilize, many studies eliminate all background tiles and make predictions on what is left [16, 19–21]. We tested whether using solely tumor tiles was sufficient for accurate predictions, and our findings were encouraging. Importantly, while it is anticipated that the tumor environment would hold the essential information for prediction, it remains unclear if surrounding tissues also carry significant information.

In the clinical setting, IHC and ISH assays remain the gold standard, however, it is important to note that a wide range of potential candidates for anti-HER2 treatment incurs screening costs and is hindered in the routine due to various reasons, such as the constrained accessibility of molecular assays, turnaround time, tissue requirements, and health plan coverage. Given that the majority of pathology slides are already routinely stained with H &E, the use of these models enables a fast and cost-effective testing in routine daily practice. For example, in cases when IHC result is 2+ “equivocal” and the HER2 status needs to be tested with additional ISH assay, the model M1 can be used to discriminate among HER2-low or HER2-high. Additionally, the M2 model might be useful for pre-screening patients aiming to detect HER2-neg vs HER2-high before the IHC assay. Finally, the M6 model could be used to discriminate HER2-neg, HER2-low or HER2-high without the need for additional tests.

While past studies have examined the ability of machine learning-based models to predict HER2 status as either positive or negative, our work attempts to expand upon the classification of these subtypes by including HER2-low as a new category. Our results are particularly relevant for two main reasons. This is the first study to detect the HER2-low biomarker from histological images considering a retrospective series of 804 patients from two distinct datasets (ACCC and HEROHE), and it elucidates the limitations of detecting HER2-low solely from IHC results [30]. Our study reveals the local cellular patterns learned for the morphological phenotypes related to the HER2-low and other subgroups, highlighting which regions of an image are important to the final prediction.

Finally, our findings further reinforce the importance of revisiting the guidelines for HER2 assessment while also supporting the inclusion of more than one pathologist in the HER2 assessment alongside the use of agreement metrics and, following the best practices for utilizing controls [7, 30, 39]. Although this practice would be ideal, many laboratories are not able to make it a reality due to the availability of pathologists and the increasing workload of these professionals.

In addition, by using H &E slides we are also investigating the potential discovery of new H &E tissue patterns that pathologists could learn from and adopt in their evaluation. Therefore, AI-based approaches, such as the one proposed in the present work, have become increasingly in demand and promise to support decision-making in the evaluation of biomarkers.

Although our results are promising, our model may fail to generalize due to overfitting, differences in imaging systems and staining protocols. Our findings may also be hampered by subjective results from IHC-based assays [30].

## Conclusion

In our retrospective study using multicentric cohort, we evaluated HER2 subgroups status using the start-of-art of deep learning models in histological images. We show that a weakly-supervised deep learning model can predict HER2 subtypes directly from H &E slides. However, we found that the performance of a model is dependent on the reliability of ground truth, which is derived from the experts interpretation of molecular assays. Future works should focus on curating datasets with high inter-expert agreement in order to mitigate subjective variability of the HER2 assessment.

### Supplementary information

Supplementary information

## Data Availability

The TCGA and HERO data used in this study are deposited in a public repository described in the Methods. We generated a dataset containing over 2 million tiles of tumor only regions, and it is publicly available at https://doi.org/10.5281/zenodo.8383580, under the Creative Commons Attribution 4.0 International license. Should there be a need for further details to reassess the data presented in this manuscript, such information can be obtained by reaching out to the primary contact upon request.

## Code Availability

All code used in training the models is available at https://github.com/tojallab/wsi-mil, under the GPLv3 license.
