# Supplementary material for: Weakly-supervised deep learning models enable HER2-low prediction from H &E stained slides
Source: Breast Cancer Res. 2024 Aug 19;26:124. doi: 10.1186/s13058-024-01863-0 (PMC11331614; doi:10.1186/s13058-024-01863-0)
Supplement: Supplementary file 1 — Supplementary information [file 13058_2024_1863_MOESM1_ESM.pdf]

Supplementary material for

Weakly-supervised deep learning models enable HER2-low prediction from H&E stained slides

Renan Valieris, Luan Martins, Alexandre Defelicibus,
Adriana Passos Bueno, Cynthia Aparecida Bueno de Toledo Osorio,
Dirce Carraro, Emmanuel Dias-Neto, Rafael A. Rosales,
Jose Marcio Barros de Figueiredo and Israel Tojal da Silva

May 17, 2024

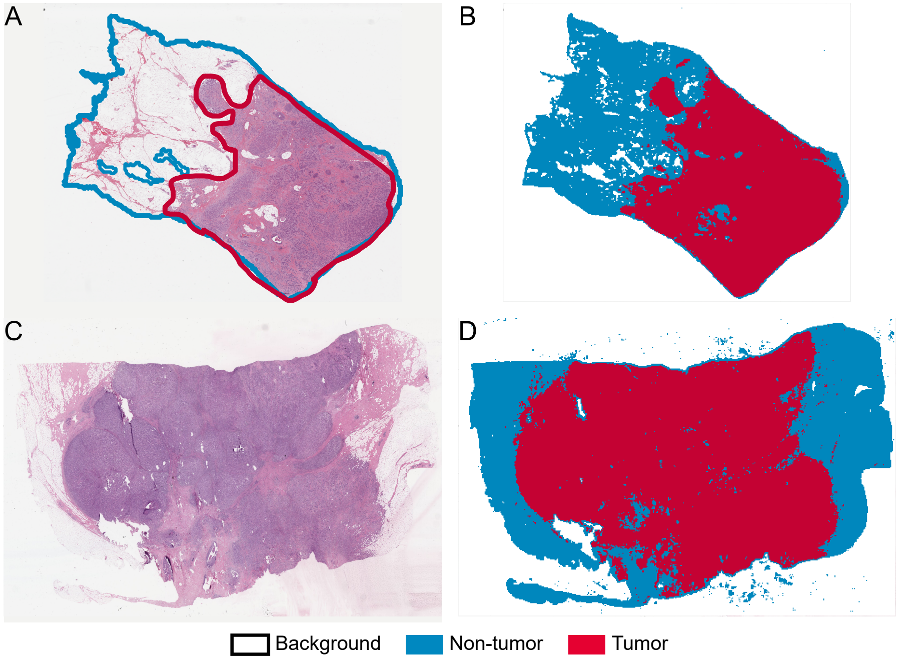

Figure 1: Tumor-background detection example. (A) Example of a slide from the TCGA-BRCA cohort with annotations of non-tumor (in blue) and tumor (in red), used to train the tumor-background detection model. (B) Model inference result on the same TCGA-BRCA slide. (C) Example of a slide from the ACCCC cohort. (D) Model inference result on the ACCCC example slide.

Table 1: Test metrics of each model on the internal and external (TCGA) sets.

Test set	Model	AUROC	Precision	Recall	F1-Score
Internal	M1	0.90 +0.01	0.82 +0.02	0.83 +0.02	0.83 +0.02
	M2	0.85 +0.02	0.77 +0.02	0.77 +0.02	0.77 +0.02
	M3	0.87 +0.02	0.81 +0.02	0.79 +0.04	0.80 +0.03
	M4	0.85 +0.01	0.70 +0.01	0.70 +0.01	0.70 +0.01
	M5	0.72 +0.02	0.65 +0.02	0.65 +0.01	0.65 +0.02
	M6	0.78 +0.02	0.57 +0.03	0.58 +0.03	0.57 +0.03
External	M1	0.63 +0.02	0.60 +0.02	0.60 +0.02	0.59 +0.03
	M2	0.79 +0.01	0.64 +0.02	0.68 +0.03	0.58 +0.04
	M3	0.62 +0.01	0.57 +0.02	0.58 +0.02	0.57 +0.02
	M4	0.65 +0.01	0.40 +0.04	0.48 +0.01	0.36 +0.02
	M5	0.63 +0.03	0.54 +0.01	0.60 +0.03	0.40 +0.02
	M6	0.61 +0.01	0.39 +0.02	0.44 +0.02	0.31 +0.02

Table 2: Histological characteristics stratified by HER2 status. SBR indicates Scarf-Bloom-Richardson. A sample is considered NEG with an IHC score of 0, LOW with an IHC score of 1+ or with an IHC score of 2+ with a negative ISH-based test result, and HIGH with an IHC score of 2+ with a positive ISH-based test or with an IHC score of 3+.

	NEG	LOW	HIGH
**Nuclear grade**			
1	5	7	1
1 and 2	2	4	0
2	79	97	11
2 and 3	2	4	3
3	113	109	121
**Inflammatory Infiltrate**			
Not found	0	2	0
Low	152	158	55
Moderate	26	29	28
High	13	2	8
**Mitotic Score**			
Score 1	104	116	42
Score 2	44	44	25
Score 3	39	27	20
**TILs**			
≥ 10	27	22	22
< 10	93	76	29
**Immunophenotype**			
Not determined	1	4	1
TNBC	43	7	0
Luminal	30	48	1
LUMA	36	20	1
LUMB	82	110	19
Luminal B-HER2	0	0	27
HER2 overexpression	0	0	46
**SBR grade**			
Grade I	26	31	1
Grade II	90	102	44
Grade III	70	53	38
**Tissue source**			
Biopsy	106	147	95
Resection	99	86	53
**Microcalcifications**			
Absent	154	153	92
Present	46	72	48
**Necrosis**			
Absent	153	167	83
Present	52	66	65
**Histological group**			
Undefined	24	27	10
In situ (DCIS)	11	36	53
No special type (NST)	134	134	71
Special type (ST)	19	18	7

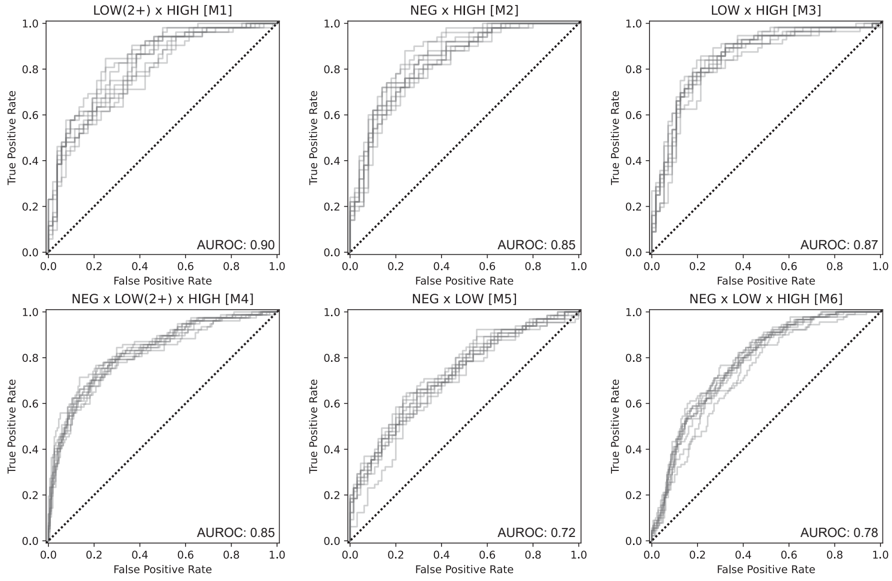

Figure 2: ROC curves obtained for each model, we plot all 10 curves obtained for each fold of the cross-validation on the internal test set. The median AUROC is noted on the bottom right.

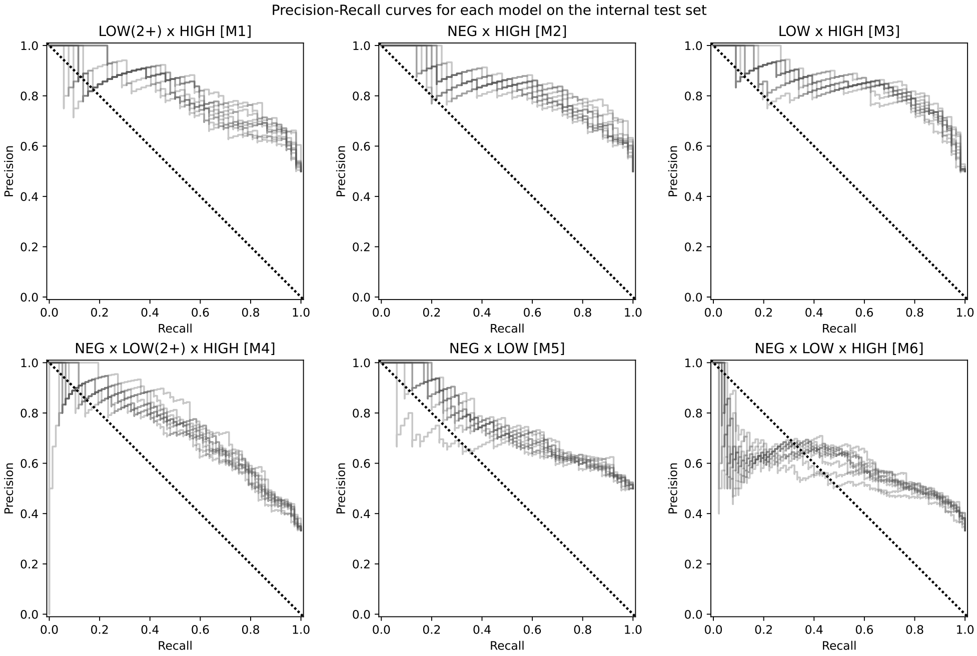

Figure 3: Precision-Recall curves obtained for each model, we plot all 10 curves obtained for each fold of the cross-validation on the internal test set.

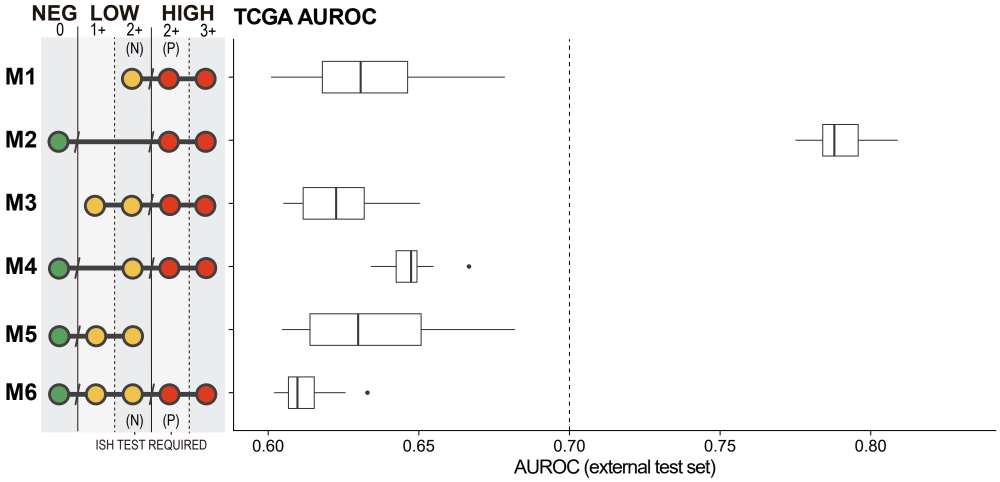

Figure 4: Model performance in the TCGA external validation set.

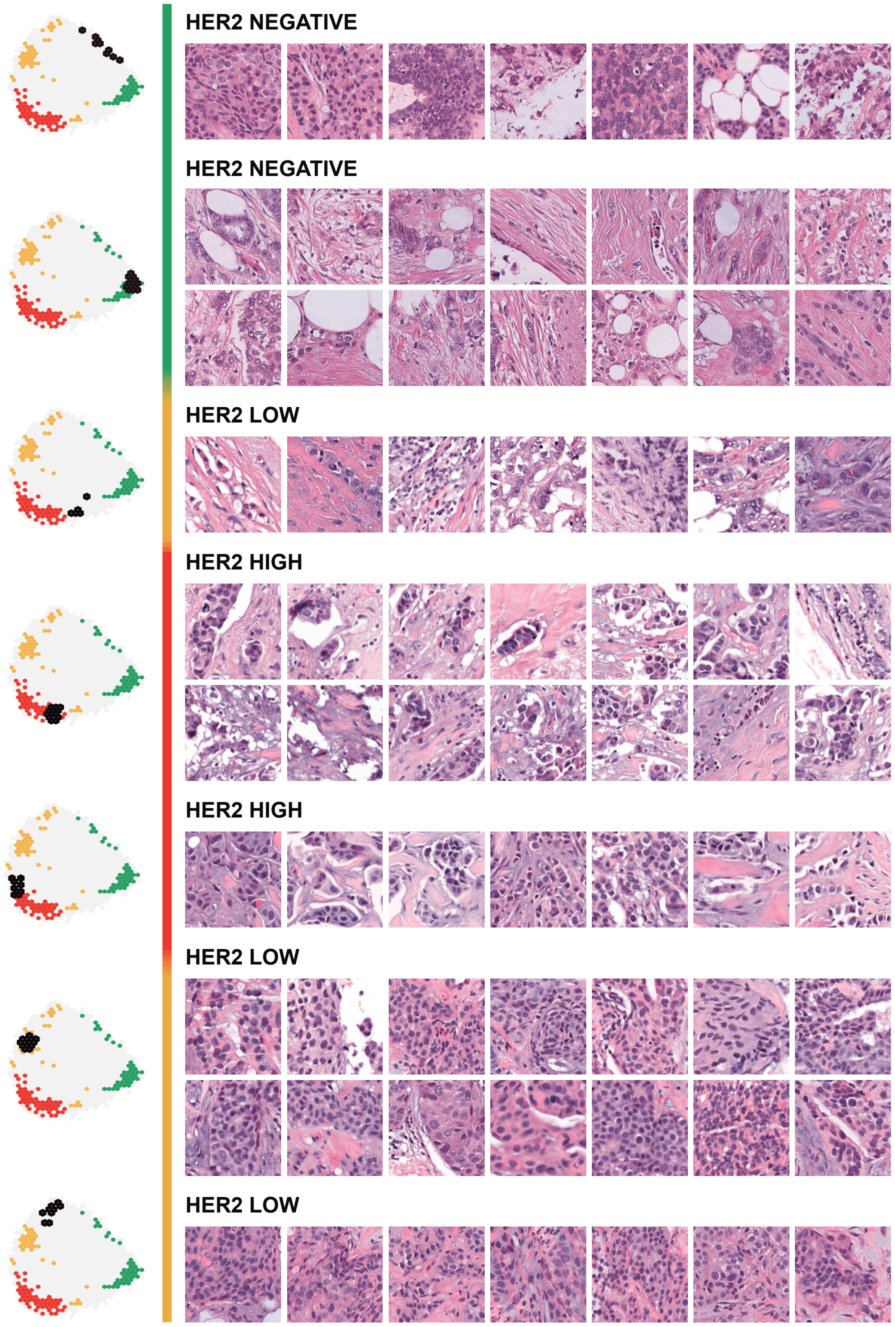

Figure 5: Examples of actionable tiles, sampled for each group from the corresponding points highlighted on the left.

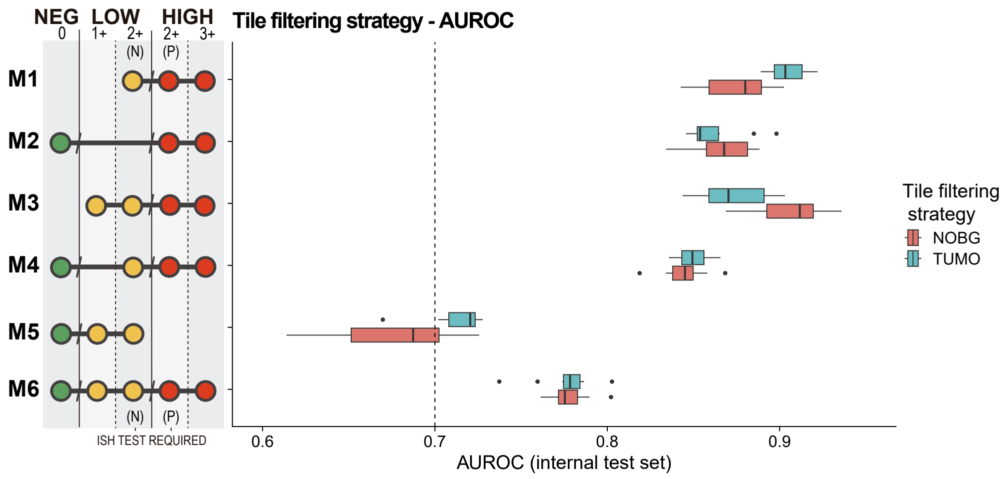

Figure 6: Internal validation test AUROC obtained with different tile filtering strategies. It can be noted that including only tiles of tumor region (TUMO) has a higher predictive performance than filtering only background tiles (NOBG).

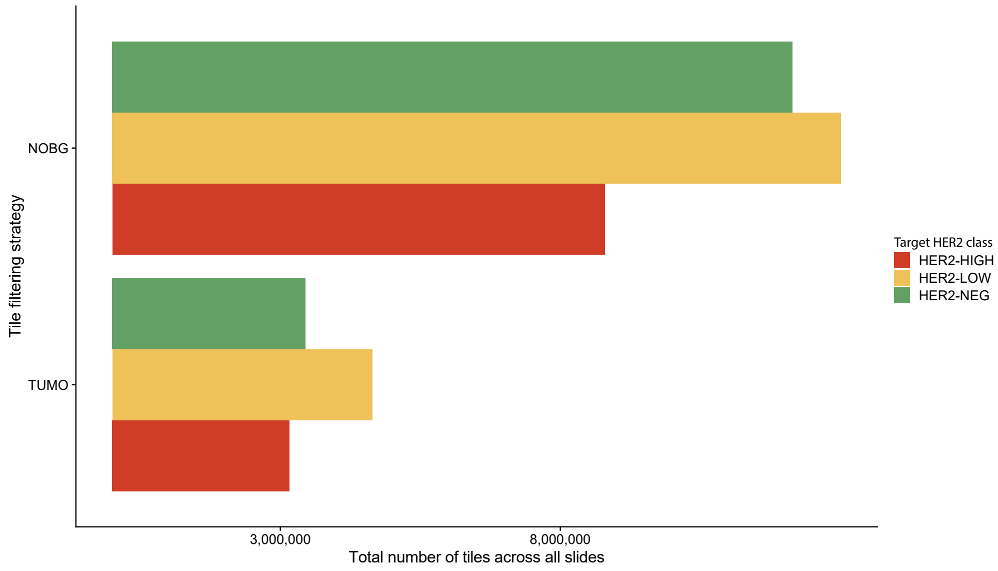

Figure 7: Number of tiles for each of the tile filtering approaches tested for each class. Filtering only background tiles leaves 3 to 5 times more tiles for training.

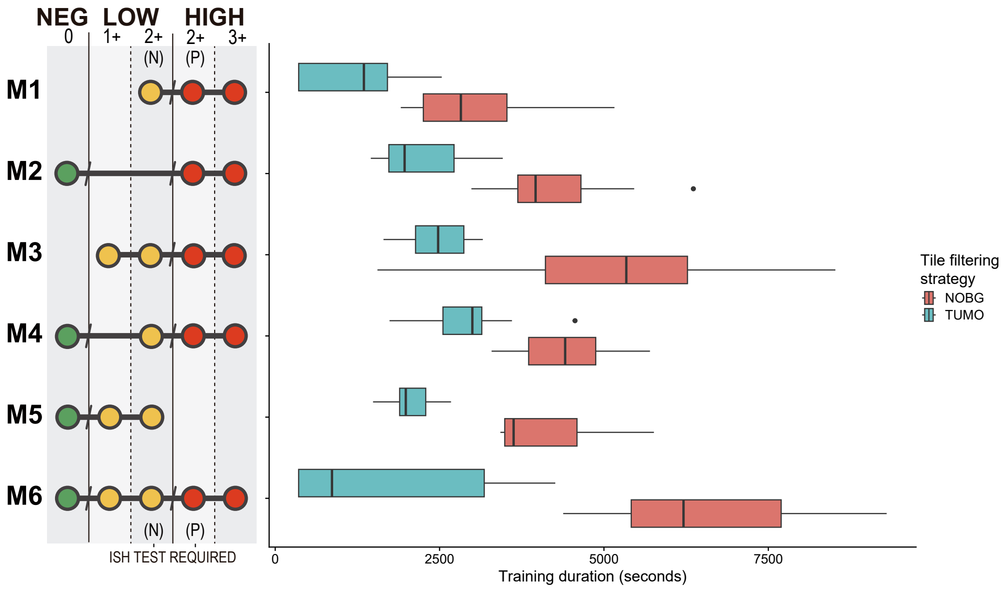

Figure 8: Training duration in seconds for different models and tile filtering strategies.

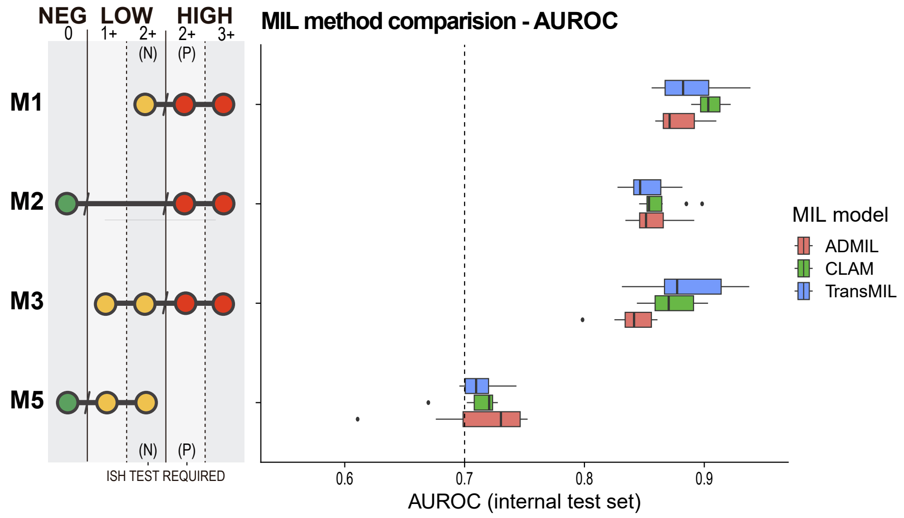

Figure 9: Performance comparison of the MIL methods on the internal test set.

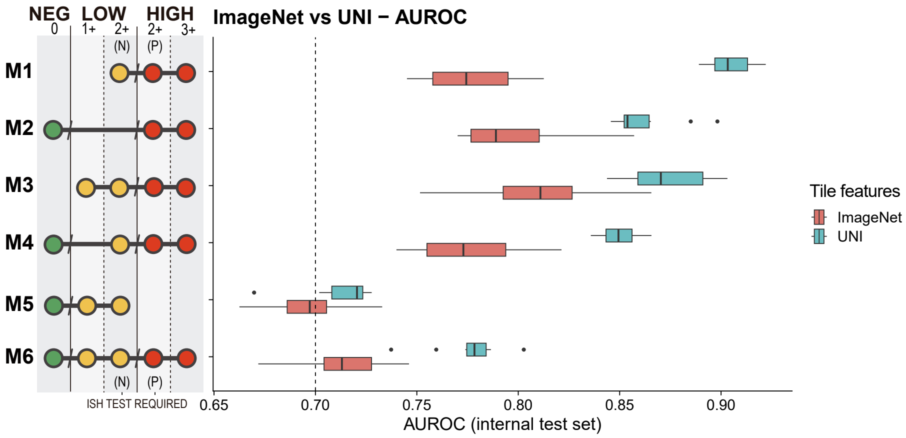

Figure 10: Internal validation test AUROC shows the performance improvement when using UNI as the feature extractor compared to an ResNet50 pretrained with ImageNet.
